# Self-assembly and adhesive properties of *Pollicipes pollicipes* barnacle cement protein cp19k: influence of pH and ionic strength

**DOI:** 10.3762/bjnano.16.129

**Published:** 2025-10-23

**Authors:** Shrutika Sawant, Anne Marie Power, J Gerard Wall

**Affiliations:** 1 Microbiology, School of Biological and Chemical Sciences, University of Galway, University Rd, Galway H91 TK33, Ireland; 2 CÚRAM Research Ireland Centre for Medical Devices, University of Galway H91 TK33, Ireland; 3 Ryan Institute, School of Natural Sciences, University of Galway H91 TK33, Ireland

**Keywords:** adhesive, amyloid fibre, barnacle cement protein, surface coating, transmission electron microscopy

## Abstract

Marine organisms such as barnacles rely on a complex underwater adhesive system, driven by self-assembly and intermolecular associations between cement proteins, for permanent attachment to a variety of surface types. In this study, we investigated the influence of environmental parameters on the self-assembly of recombinant cp19k, a key adhesive protein in *Pollicipes pollicipes*. Using TEM imaging, a low pH (4.0) and high salt concentration (600 mM NaCl) environment, mimicking *P. pollicipes* gland conditions, was identified to promote the formation of extended, needle-like fibrils by the cp19k protein. The β-amyloid nature of fibrils formed under these conditions and at high pH/low salt concentration was confirmed by Thioflavin T assay. Non-fibrillar cp19k adhered most effectively to hydrophilic and hydrophobic surfaces under low pH/low salt concentration conditions, while pre-formed fibrils retained their adhesion ability upon switching to a high pH/high salt concentration environment, which was designed to mimic the change in the protein environment upon secretion in vivo. These findings support the hypothesis that fibril formation occurs in the acidic, iso-osmotic gland of the barnacle, with delayed cement curing enabling fibril secretion for sustained adhesion of the organism. The study provides insight into the environmental sensitivity of cp19k structure–function dynamics and may support the design of bioinspired adhesives and biomaterials.

## Introduction

Marine adhesives are naturally occurring substances secreted by a variety of organisms to attach themselves to submerged surfaces such as rocks, ship hulls, and even other organisms [1]. These bioadhesives function under challenging aquatic conditions, including high and fluctuating salinity, and constant turbulence. Unlocking the molecular mechanisms behind their ability to achieve robust, long-term adhesion under wet and dynamic conditions may help to inform the design of eco-friendly adhesives for application in biomedicine, industry, and underwater engineering [2–3].

Mussel adhesive proteins are the most extensively studied of marine bioadhesives. Mussels anchor to submerged surfaces using a byssus, a bundle of proteinaceous threads secreted by the foot [4]. Each thread ends in an adhesive plaque composed of mussel foot proteins (Mfps), which are rich in the modified amino acid ʟ-3,4-dihydroxyphenylalanine (DOPA) [5]. DOPA is formed via post-translational hydroxylation of tyrosine and mediates wet surface adhesion through hydrogen bond formation, metal chelation, and covalent interactions [6]. The byssal threads display a mechanical gradient, with regions proximal to the animal more elastic and distal regions stiffer and tougher [7]. This design allows for effective dissipation of hydrodynamic forces and maintains attachment of the threads under wave-induced stress, thereby allowing the byssus to function as both an adhesive and a shock-absorbing tether [7].

Mussel adhesives are difficult to re-create either synthetically or recombinantly [8], the latter due largely to difficulties associated with post-translational modifications in recombinant protein expression systems [9]. Cell-Tak™ is a commercial mixture of mussel foot proteins Mfp-1 and Mfp-2 from *Mytilus edulis* [10] but purification of Mfp-based adhesives necessitates harvesting and chemical processing of large quantities of mussels, raising ecological and scalability concerns [10]. Additionally, *M. edulis* Mfp exhibits optimal adhesion under acidic conditions [11], potentially limiting its biomedical application. Meanwhile, synthetic sealants based on DOPA functionalisation of natural or synthetic polymers have shown promise in biomedical applications, but their formulation, biocompatibility, long-term stability, and clinical efficacy still require significant investigation [12–13].

Barnacles are sessile marine organisms which employ a different strategy for underwater adhesion: they secrete a multicomponent proteinaceous cement that facilitates robust, permanent attachment to a wide range of natural and synthetic surfaces [1,14–15]. Notably, the absence of DOPA means that several barnacle cement proteins have been produced in *E. coli*, providing an advantage over mussel adhesive proteins in terms of reproducibility and scalability [16–22].

Studies on barnacle cement proteins have predominantly focused on acorn barnacles with calcareous (calcium carbonate) bases (e.g., *Balanus albicostatus*) to date. The goose barnacle *Pollicipes pollicipes* is a sessile crustacean with body parts encased in calcified plates and a cuticle-covered stalk (peduncle) that anchors it to a surface [23]. Structural and biochemical analyses revealed that the cuticle is primarily composed of α-chitin, with indications of elastin-like proteins and collagen [23], and stiffness values comparable to those found in elastomers and in the soft cuticles of crustaceans following molting [23]. This flexibility of the cuticle allows for attachment in environments where rigid adhesion would fail, such as on soft tissues, polymers, or dynamic interfaces [23–24]. Stalked barnacles exhibit significant evolutionary divergence (200–250 million years) from acorn barnacles [25] and inhabit different ecological niches, suggesting potentially distinct biochemical properties in their adhesive systems [14]. Most functional studies on *P. pollicipes* cement proteins to date have been limited to in silico analyses [26–27] or basic characterisation under seawater-like (basic pH with high salt concentration) or proposed gland-like (acidic pH with low salt concentration) conditions [28–31], however, with much still unknown about their structural properties or mechanical function under varied environmental conditions.

Among *P. pollicipes* cement proteins, cp19k has emerged as a key contributor to underwater adhesion [16,32]. We previously described recombinant production of the 19 kDa *P. pollicipes* cement protein (rPpolcp19k) and its adhesion on various substrate chemistries [21]. The protein self-assembled into intertwined amyloid fibres [22] and has the potential to form amyloid-like fibrillar aggregates, a structural motif increasingly implicated in barnacle adhesion [33–34]. Amyloid fibres are characterised by their β-sheet-rich architecture and have been linked to increased cohesive strength and durability in marine adhesives [35–36].

In the present work, we expressed recombinant *P. pollicipes* cp19k (rPpolcp19k) in *E. coli* and identified key environmental modulators of fibril formation by the protein. Transmission electron microscopy (TEM) was used to study the rate of fibril formation and morphology under varied pH and salt concentration conditions, while Thioflavin T was utilised to detect β-sheet content and provide insight into the amyloidogenic nature of the assembled structures. Finally, the adhesive properties of monomeric and fibrillar rPpolcp19k-his were investigated to better understand the relationship between structural conformation and adhesion in the protein.

## Results

### Protein expression and fibril formation

Co-expression of rPolcp19k-his with *E. coli* GroEL-GroES chaperones to improve folding was performed as described previously [21]. Purified rPolcp19k-his protein yields of 1.8 to 2 mg per litre of *E. coli* culture were achieved (Supporting Information File 1, Figure S1). Purified protein was dialysed into 10 mM sodium acetate (pH 4.0) or 10 mM sodium phosphate (pH 8.0) buffer, with NaCl concentrations ranging from 0 to 600 mM.

#### Transmission electron microscopy

**Figure 1:**
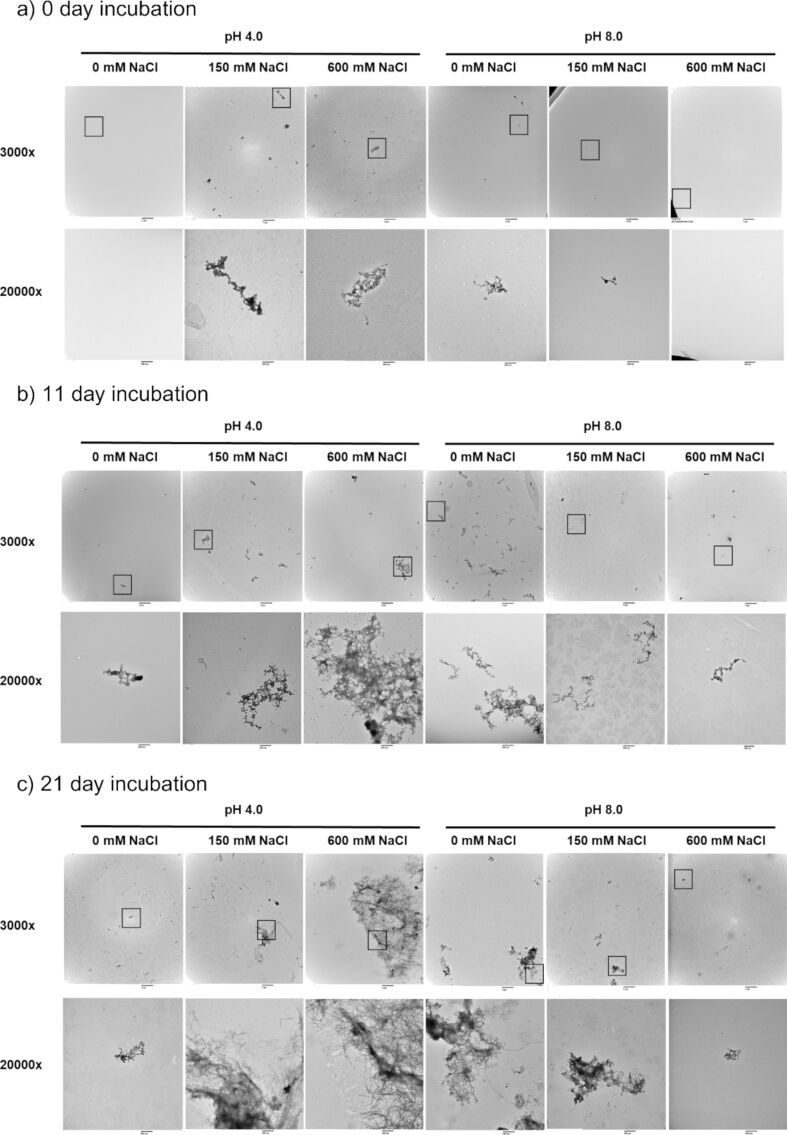
TEM images of rPolcp19k-his protein samples after incubation under the indicated pH and salt concentration conditions for (a) 0 days, (b) 11 days, and (c) 21 days. Squares in 3000× magnification images represent areas shown at 20000× magnification in the corresponding panels below. The scale bar represents 4 µm (3000× magnification images) or 600 nm (20000× magnification images).

**Figure 2:**
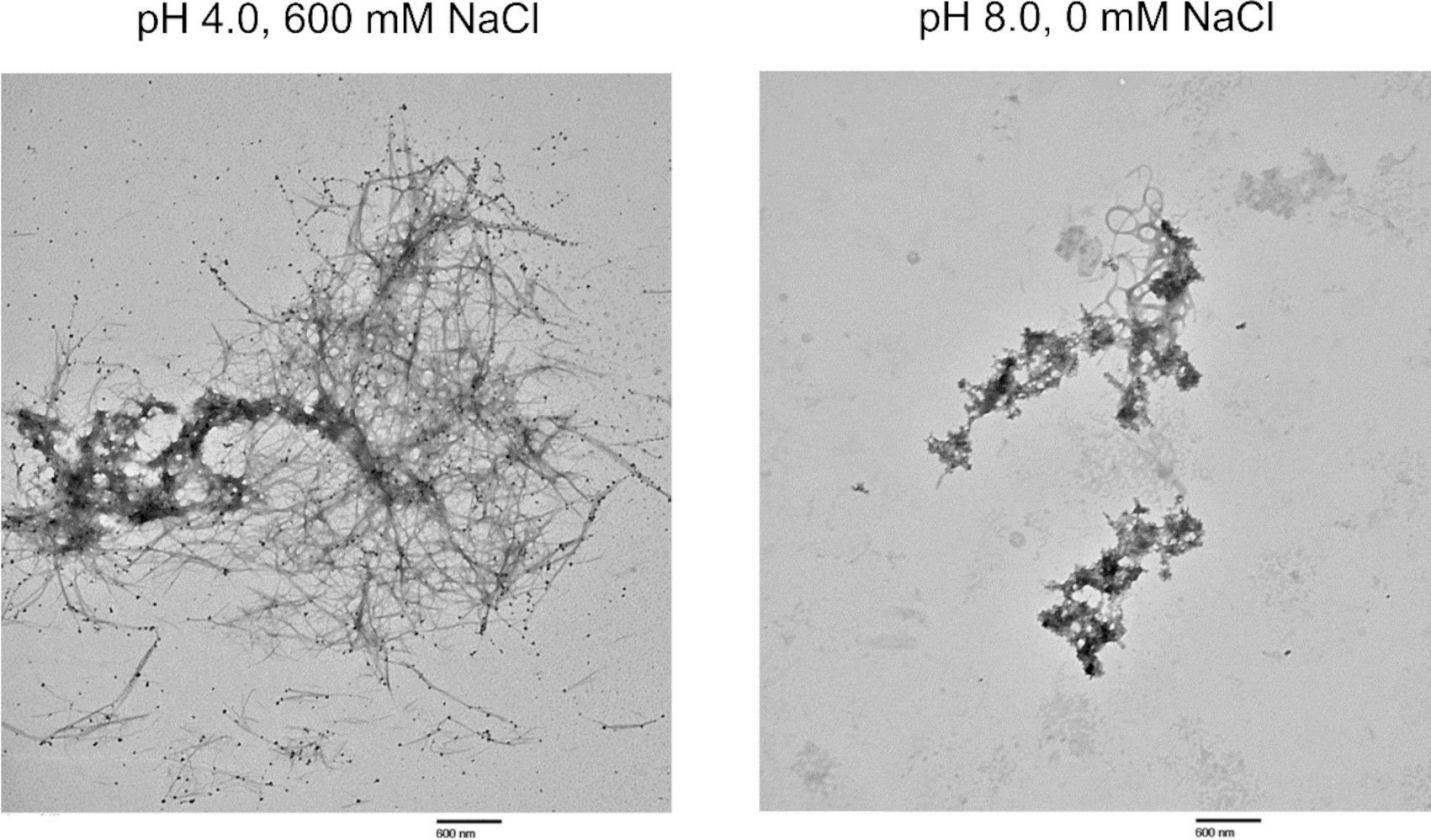
TEM images of rPolcp19k-his protein samples after incubation for 21 days. Fibres formed under pH 4.0, 600 mM NaCl (left) and pH 8.0, 0 mM NaCl (right) conditions, at 20000× magnification are shown. The scale bar represents 600 nm.

TEM analysis identified the formation of fibril structures by rPolcp19k-his under a variety of conditions analysed. Fibre networks were clearly more extensive in the presence of low pH/high salt concentration and high pH/low salt concentration than under the other tested conditions. Fibrils appeared as early as day 0 (Figure 1a) or day 3 (Supporting Information File 1, Figure S2) in these samples and became more abundant by day 11 (Figure 1b). Fibril formation was most pronounced at pH 4.0 in the presence of 600 mM NaCl, with highly abundant and well-dispersed structures clearly visible throughout the sample after 21 days of incubation (Figure 1c). By day 21 also (Figure 1c), apparent morphological differences were evident between fibrils formed at pH 4.0 (needle-like and extended) and those formed at pH 8.0 (more coiled and entangled) (Figure 2).

#### Thioflavin (ThT) binding assay

**Figure 3:**
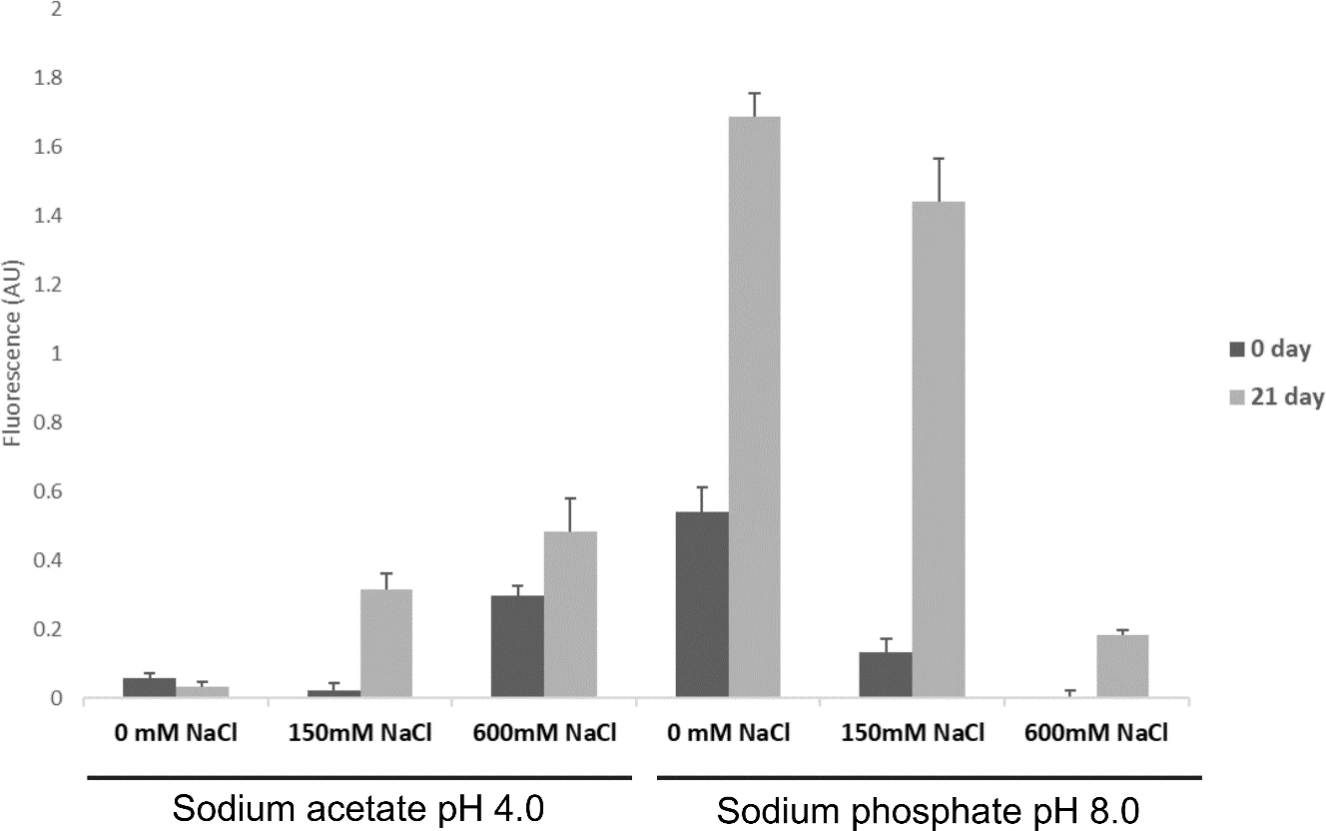
ThT assay analysis of rPolcp19k-his protein samples incubated for 0 and 21 days in the indicated buffers. Assay results are presented in arbitrary units (AU) of fluorescence and as mean ± standard deviation; *n* = 3.

The ThT assay showed the greatest increase in fluorescence intensity over 21 days in rPolcp19k-his protein samples that had been incubated at pH 8.0 with no NaCl, followed by rPolcp19k-his at pH 4.0 and 600 mM NaCl or 150 mM NaCl (Figure 3). This is indicative of the occurrence of β-amyloid in the samples and, combined with the TEM analysis (Figure 1 and Figure 2), indicates that the cp19k protein forms amyloid fibres, potentially with diverse morphologies, under a variety of physicochemical conditions. Lysozyme and BSA (negative controls) and heat-denatured lysozyme (positive control) were used to validate the assay (Supporting Information File 1, Figure S3).

#### Adhesion analysis

**Figure 4:**
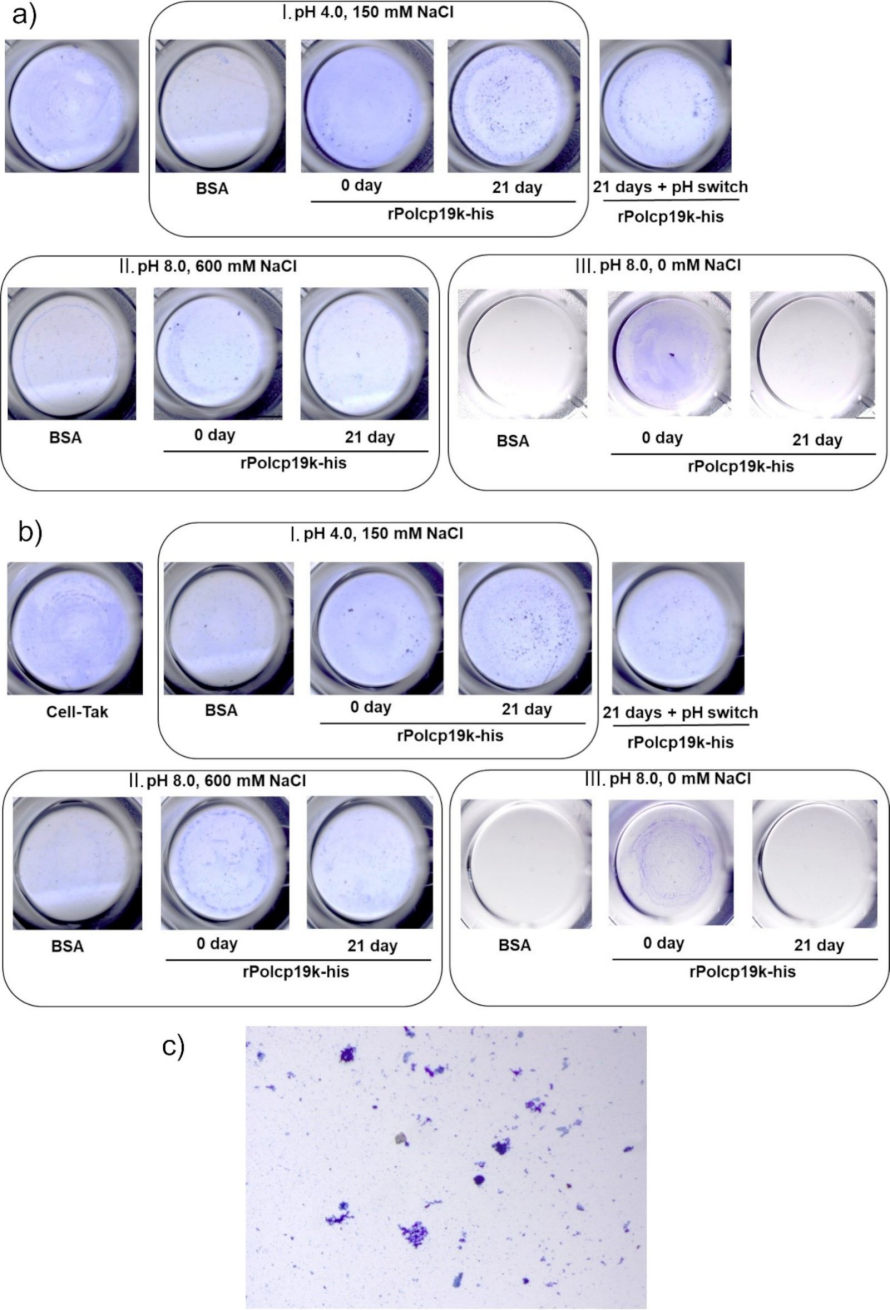
Analysis of adhesion of rPolcp19k-his protein to (a) hydrophilic and (b) hydrophobic polystyrene surfaces in the presence of (I.) pH 4.0, 150 mM NaCl; (II.) pH 8.0, 600 mM NaCl; and (III.) pH 8.0, 0 mM NaCl. 21 days + pH switch: rPolcp19k-his incubated in pH 4.0, 150 mM NaCl for 21 days, followed by switching to pH 8.0, 600 mM NaCl prior to adhesion analysis. Wells are 6.5 mm in diameter, viewed at 6.5× magnification. 0 day: protein samples were not pre-incubated for fibre formation before adhesion analysis. 21 day: protein samples were pre-incubated under the indicated conditions for 21 days before adhesion analysis. (c) rPolcp19k-his incubated at pH 4.0, 150 mM NaCl for 21 days, on hydrophilic surface, at 11.5× magnification.

rPpolcp19k-his protein samples were analysed for adhesion after prior incubation for 21 days under a variety of pH and salt concentration conditions to allow for fibre formation, or without any pre-incubation. Protein samples that had not been incubated prior to the adhesion assay to enable fibre formation (0 day) demonstrated relatively homogeneous staining on both hydrophilic and hydrophobic materials, indicating adhesion to the relevant surfaces (Figure 4a,b). Staining was noticeably more intense for protein samples incubated at pH 4.0 and 150 mM NaCl, followed by samples incubated at pH 8.0 and 0 mM NaCl (Figure 4a,b). Additionally, denser staining in a heterogenous, granulated pattern, thought to correspond to clusters of aggregated proteins, was observed to occur on both surfaces in samples that had been incubated for 21 days at pH 4.0, 150 mM NaCl (Figure 4c), whereas these were not evident in either pH 8.0 environment. No significant change in staining was observed when samples that had been incubated for 21 days at pH 4.0, 150 mM NaCl to allow fibres to form were switched to pH 8.0, 600 mM NaCl (to mimic seawater) prior to carrying out the adhesion analysis (Figure 4a,b).

## Discussion

This study investigates the physicochemical conditions of fibril formation by recombinant *P. pollicipes* cp19k, a key protein involved in barnacle underwater adhesion. The results identify a combination of low pH and high salt concentration as optimal for fibril formation, whereas previous reports with cp19k homologues from other barnacle species reported that fibres predominantly formed under either “gland-like” (low pH, low salt concentration) [29] or seawater-mimicking (high pH, high salt concentration) conditions [31].

We hypothesise that the combination of low pH and high ionic strength mimics the osmotic conditions pertaining in the *P. pollicipes* adhesive glands. As barnacles are osmoconformers rather than osmoregulators [37], their internal fluid environment is iso-osmotic to seawater. While species such as *Balanus improvisus* can adapt to brackish conditions, *P. pollicipes* is an open-coast barnacle optimally adapted to full-strength seawater [38]. Thus, although the adhesive glands are acidic [14,39], their osmolarity is likely comparable to that of seawater. Our findings suggest, therefore, that fibril formation is favoured in the gland environment in *P. pollicipes*. As this process is slow, apparently requiring days to several weeks, this likely prevents premature cement curing within the gland – this is the post-secretion process of enzymatic and oxidative crosslinking of adhesive proteins to transform them into a hardened, insoluble matrix that enables permanent underwater adhesion of the barnacle [1].

While fibrils also formed under high pH and low salt concentration conditions in this study, these appeared more curled and tangled than the extended, needle-like conformations observed in the low pH/high salt concentration environment. Both fibril forms (needle-like and curled) tested positive in the ThT assay, indicating that, despite the difference in morphology, they contain a β-sheet-rich architecture characteristic of amyloid structures, which is increasingly implicated in enhancing cohesive strength in marine adhesives [33]. rPolcp19k-his fibrils formed in the high pH/low salt concentration environment exhibited the highest fluorescence, though more detailed investigation using methods such as attenuated total reflectance-Fourier transform infrared (ATR-FTIR) spectroscopy or circular dichroism (CD) will be necessary to confirm an elevated β-sheet content in these fibrils. A similar curled-fibril morphology has been reported for cp19k homologues from other barnacle species, but at the low pH/low salt concentration conditions noted above, and these fibrils did not exhibit fluorescence in ThT assays and were instead proposed to be largely non-amyloid and α-helical in content [29]. Moreover, no fibrils were observed at pH 8.0–9.9, 150 mM NaCl in the previous study [29], in contrast with our present observations, though the authors did not investigate the pH 4.0, 600 mM NaCl conditions in which fibril formation was most pronounced in our work [29–31].

A study of a recombinantly produced *Balanus albicostatus* cp19k protein from which cysteine residues had been removed reported the formation of fibrils only under seawater-mimicking conditions (pH 8.0, high salt concentration) and not in an acidic environment [31]. While the modified amino acid sequence may have impacted the protein morphology, the differences from both other published results with cp19k homologues [29] and the present study underscore the importance of protein construct design as well as environmental parameters in determining the self-assembly behaviour of cp19k.

We also investigated the adhesive properties of the rPpolcp19k-his protein, in its unassembled and fibrillar forms. The unassembled or 0-day incubated protein molecules exhibited greater and homogenous adhesion in a low pH/low salt concentration than in a high pH/high salt concentration environment, which is consistent with a previous report that monomeric cp19k lost adhesion at high pH/high salt concentration [29]. The same authors reported that pre-assembled aggregates of cp19k were more stable and exhibited enhanced adhesion at high pH [29], while in our work, samples that were incubated for 21 days under native gland-like conditions forming thin needle like fibres were observed to form clusters of apparently aggregated protein which exhibited surface binding. This supports the hypothesis that unstructured or partially assembled cp19k fibrils may maximise surface interaction prior to curing and becoming stained by Coomassie homogenously, whereas fibrillar aggregates formed under gland-like conditions, though structurally robust, may offer less surface contact, resulting in the granular Coomassie staining. The curled fibres formed in low pH and low salt concentration conditions were also tested for surface adhesion, resulting in no homogenous or even no granular staining. We hypothesise that these curled fibres formed under non-native like conditions hold no adhesion value.

In order to mimic the natural process by which cp19k protein is synthesised and possibly forms fibrils in a low pH/high salt concentration gland environment, before secretion into the higher pH/similar salt concentration seawater, rPpolcp19k-his samples that had undergone assembly into fibrils for 21 days at pH 4.0, 150 mM NaCl were transferred into a pH 8.0, 600 mM NaCl environment prior to investigation of adhesion. Similar aggregates of proteinaceous material were observed before and after the pH switch, and no observable difference in adhesion was noted, suggesting that cp19k fibrils remain stable and retain their adhesion ability after the pH switch. To further correlate these observations with the natural life cycle of barnacle adhesion, more detailed AFM- or quartz crystal microbalance-dissipation (QCM-D)-based studies of the adhesive properties of rPolcp19k-his monomers and fibres formed under different physicochemical conditions, as well as after a similar pH switch, are planned.

## Conclusion

This study identifies a low pH and high salt concentration environment as optimal for formation of β-amyloid-containing fibrils by recombinant *P. pollicipes* cp19k (rPpolcp19k). We hypothesise that these conditions reflect the acidic, iso-osmotic environment of the adhesive glands in *P. pollicipes*, an open-coast species adapted to full-strength seawater. The different morphologies and β-amyloid content of fibrils formed under different pH and salt concentration conditions demonstrate the conformational plasticity of cp19k in response to its environment, with its self-assembly into elongated fibrils favoured under low pH/high salt concentration conditions, and the prolonged timescale of fibrillogenesis likely preventing premature adhesive curing within the gland prior to secretion. Overall, our findings support a model in which cp19k undergoes pH- and salt concentration-dependent self-assembly into fibrils in the cement gland, with implications for the mechanism and timing of underwater curing in barnacle bioadhesion. While monomeric cp19k exhibited apparently stronger surface adhesion than fibrillar protein, differences in structure, β-amyloid composition, and adhesive properties between the monomeric and the fibrillar protein, as well as between fibrils assembled under different conditions, will be further investigated through a detailed macro- and nanoscale study designed to understand the relationship between structural transitions and function of the protein in vivo.

## Experimental

### Recombinant protein expression and purification

rPpolcp19k-his protein was expressed in *E. coli* BL21 (DE3) cells as previously described [21] and purified by two step purification, that is, immobilised metal affinity chromatography (IMAC) followed by ion exchange chromatography (IEC). For IMAC, the Co-IDA resin column was washed with 25 mM Tris-HCl buffer (pH 8.0) containing 150 mM NaCl and 0.1% Triton, and eluted using the same buffer containing 150 mM imidazole. Elution fractions containing rPpolcp19k-his protein were pooled and dialysed against 25 mM Tris-HCl (pH 8.0) buffer overnight at 4 °C and passed through an ion exchange UNO-S resin that had been equilibrated with 25 mM Tris-HCl (pH 8.0). The column was washed using equilibration buffer containing 100 mM NaCl, and bound protein was eluted in equilibration buffer containing 300 mM NaCl. Eluted fractions from both columns were analysed by SDS-PAGE and immunoblotting, as described in Supporting Information File 1, Figure S1.

### Formation of protein fibrils

Purified rPpolcp19k-his protein was concentrated to 500 µg/mL using an Amicon^®^ Ultra Centrifugal Filter, 10 kDa MWCO (Merck). The protein was reconstituted at 500 µg/mL in 10 mM sodium acetate buffer (pH 4.0) or 10 mM sodium phosphate buffer (pH 8.0) with NaCl concentrations from 0–600 mM, using a Slide-A-Lyzer™ MINI dialysis device, 10K MWCO (Thermo Fisher Scientific). Samples were incubated at 25 °C for up to 21 days, removed at intervals, snap frozen in liquid nitrogen, and stored at −80 °C for further analysis

### Transmission electron microscopy

A 10 µL aliquot of rPpolcp19k-his protein (500 µg/mL), incubated for 0, 3, 11, or 21 days under varying pH and NaCl conditions, was applied to 200 mesh Cu formvar/carbon-coated grids (Agar Scientific) and allowed to settle for 5 min. Grids were washed three times in phosphate-buffered saline for 5 min each, followed by three washes in dH_2_O. Negative staining with uranyl acetate was performed by incubating 5 µL of R1000 UA-Zero EM stain (Agar Scientific) on grids for 3 min. Grids were washed five times with dH_2_O and air-dried overnight. Bright-field TEM imaging was carried out using a Hitachi H7000 microscope operated at an accelerating voltage of 80 kV, and images were acquired at 3000× and 20000× magnifications. Each sample was initially scanned throughout at 3000× magnification to identify proteinaceous material; 10–15 TEM fields were then captured at 20000× magnification, and representative images containing protein, if present, were selected. Analysis of pH 8.0, 600 mM NaCl 21 day, pH 8.0, 0 mM NaCl 21 day, pH 8.0, 0 mM NaCl 11 day, and pH 4.0, 150 mM NaCl 11 day samples was repeated to confirm initial observations.

### ThT

ThT, a dye that selectively binds to β-sheet-rich regions in amyloid fibrils and exhibits enhanced fluorescence with a redshift in emission upon binding [5], was used to investigate β-amyloid fibril formation by the rPpolcp19k-his protein. Protein samples (0.5 mg/mL) were incubated at 25 °C under varying pH and NaCl conditions, and analysed after 0 and 21 days for the presence of amyloid. Protein samples (100 µL) were mixed with 100 µL of 40 µM ThT prepared in the sample buffer at the same pH and incubated at room temperature for 5 min. Fluorescence emission was recorded at 482 nm using a Varioskan Flash spectrofluorometer in a black polystyrene 96-well plate, with excitation at 440 nm and a bandwidth of 12 nm. Spectra were plotted after subtracting the fluorescence values of the respective buffers. Negative controls (bovine serum albumin (BSA; lyophilised powder, Merck) and hen egg white lysozyme (Fluka)) and a positive control (hen egg white lysozyme denatured at 60 °C for 48 h in 0.1 M HCl-KCl buffer, pH 2.0) were included in the assay.

### Surface adhesion assay

The adhesion of rPpolcp19k-his in both monomeric and fibril form was studied by surface coating assay using surfaces typical of cell culture experiments, namely, hydrophilic 96-well polystyrene tissue culture-treated plates (Sarstedt), and hydrophobic Nunc™ MicroWell™ 96-well untreated plates (Thermo Scientific™). Cell-Tak (Corning^®^ Cell-Tak™, Fisher Scientific), containing a mixture of *Mytilus edulis* adhesive proteins mfp-1 and -2, and BSA were used as positive and negative controls, respectively.

Cell-Tak was prepared in 5% acetic acid according to the manufacturer’s instruction and investigated at 10 µg/cm^2^. Unassembled rPpolcp19k-his and rPpolcp19k-his incubated for 21 days for fibril formation and BSA were coated at 30 µg/cm^2^. In addition, rPpolcp19k-his samples incubated for 21 days at pH 4.0, 600 mM NaCl and subsequently switched to pH 8.0, 600 mM NaCl were analysed at 30 µg/cm^2^ as above.

BSA was tested under the same conditions as rPpolcp19k-his. Cell-Tak was mixed with a threefold volume of 0.1 M sodium bicarbonate buffer (pH 8.3), according to the manufacturer’s instructions, followed by incubation in wells at 25 °C for 48 h. After all incubations, the protein solution was aspirated, wells were washed three times with dH_2_O water for 5 min each, and Coomassie Blue stain was added for 15 min, followed by three destaining steps for 5 min each. Experiments were performed in triplicate, and wells were viewed on an Olympus SZX16 Stereo Microscope at 6.5× magnification.

## Supporting Information

File 1 Additional figures.

## Data Availability

Data generated and analyzed during this study is available from the corresponding author upon reasonable request.
