# Supplementary material for: Self-assembly and adhesive properties of Pollicipes pollicipes barnacle cement protein cp19k: influence of pH and ionic strength
Source: Beilstein J Nanotechnol. 2025 Oct 23;16:1863–72. doi: 10.3762/bjnano.16.129 (PMC12557431; doi:10.3762/bjnano.16.129)
Supplement: File 1 — Additional figures. [file Beilstein_J_Nanotechnol-16-1863-s001.pdf]

BEILSTEIN JOURNAL OF NANOTECHNOLOGY

Supporting Information

for

Self-assembly and adhesive properties of *Pollicipes pollicipes* barnacle cement protein cp19k: influence of pH and ionic strength

Shrutika Sawant, Anne Marie Power and J. Gerard Wall

Beilstein J. Nanotechnol. **2025,** *16,* 1863–1872. doi:10.3762/bjnano.16.129

Additional figures

License and Terms: This is a supporting information file under the terms of the Creative Commons Attribution License (https://creativecommons.org/licenses/by/4.0). Please note that the reuse, redistribution and reproduction in particular requires that the author(s) and source are credited and that individual graphics may be subject to special legal provisions.
The license is subject to the *Beilstein Journal of Nanotechnology* terms and conditions: (https://www.beilstein-journals.org/bjnano/terms)

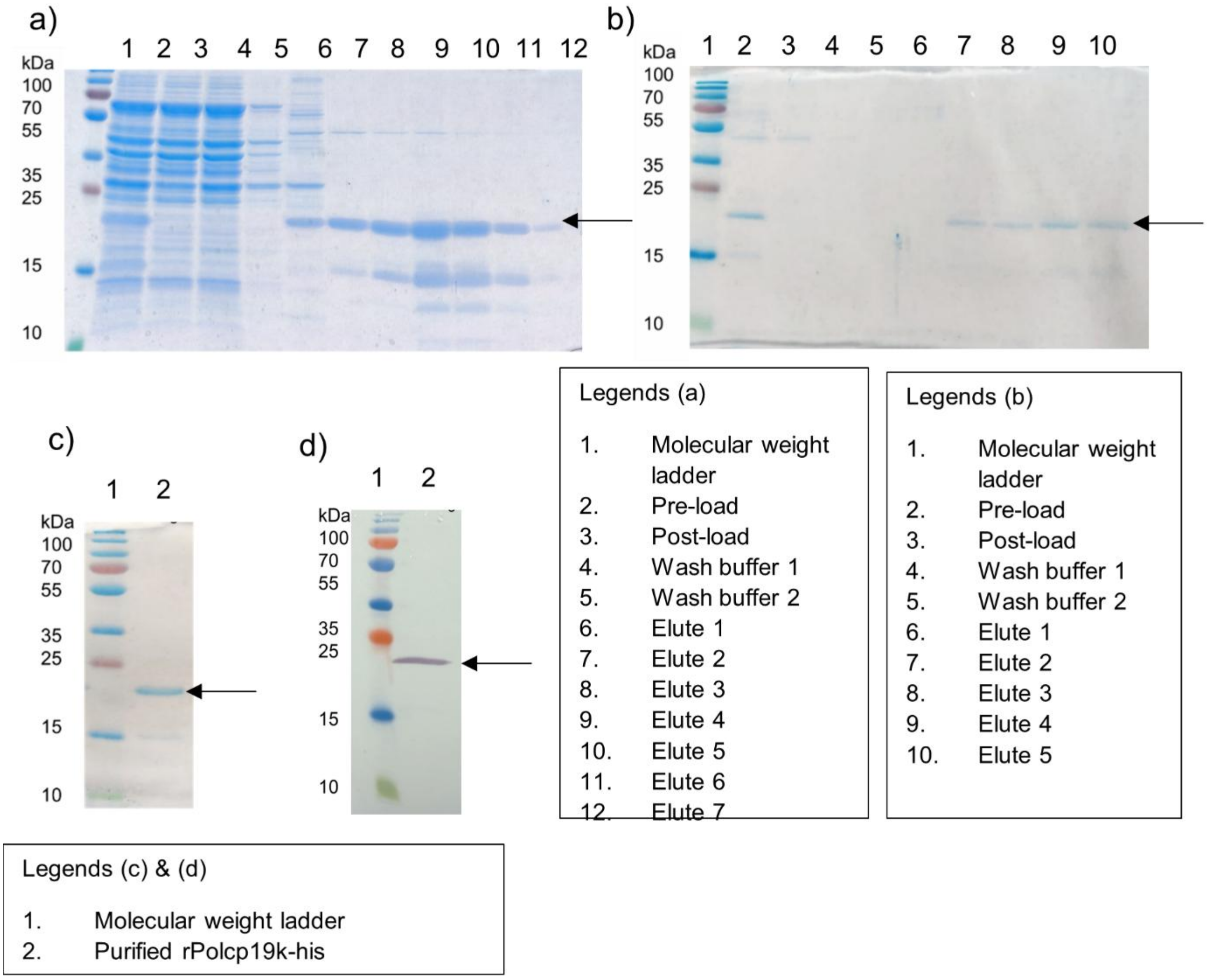

Figure S1: Expression and purification of rPolcp19k-his. SDS-PAGE analysis of a) IMAC and b) IEC purification of rPolcp19k-his. c), d): SDS-PAGE and Western blot analyses, respectively, of purified protein. Arrows indicate proteins of the expected size of pPolcp19k-his.

Protein samples were analysed under denaturing conditions in 15% SDS-PAGE. Gels were stained with InstantBlue® Coomassie Protein Stain (Abcam) or proteins were transferred to an Amersham Protran™ 0.2 µm nitrocellulose blotting membrane (GE Healthcare, Fischer scientific) for immunodetection of rPpolcp19k-his using a monoclonal anti-polyhistidine peroxidase-conjugated antibody (Sigma Aldrich, Ireland) diluted 1:1000 in Tris-buffered saline (25 mM Tris, pH 7.4, 150 mM NaCl). Colour was developed using Tetramethylbenzidine (TMB).

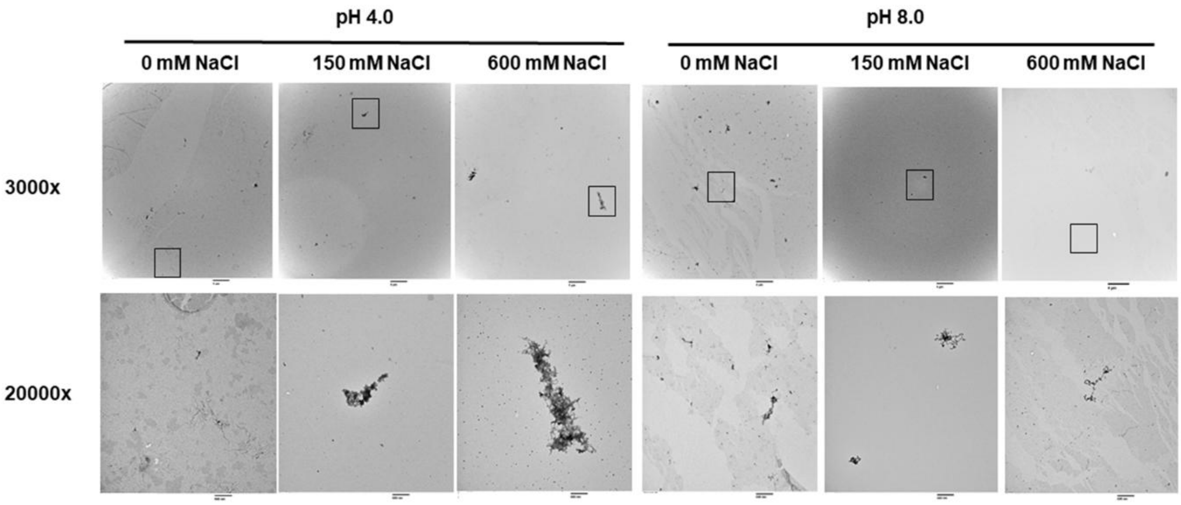

Figure S2: TEM images of rPolcp19k-his protein samples after incubation for 3 days under the indicated pH and salt conditions. Squares in 3000x magnification images represent areas shown at 20000x magnification in the corresponding panels below. Scale bar represents 4 µm (3000x magnification images) or 600 nm (20000x magnification images).

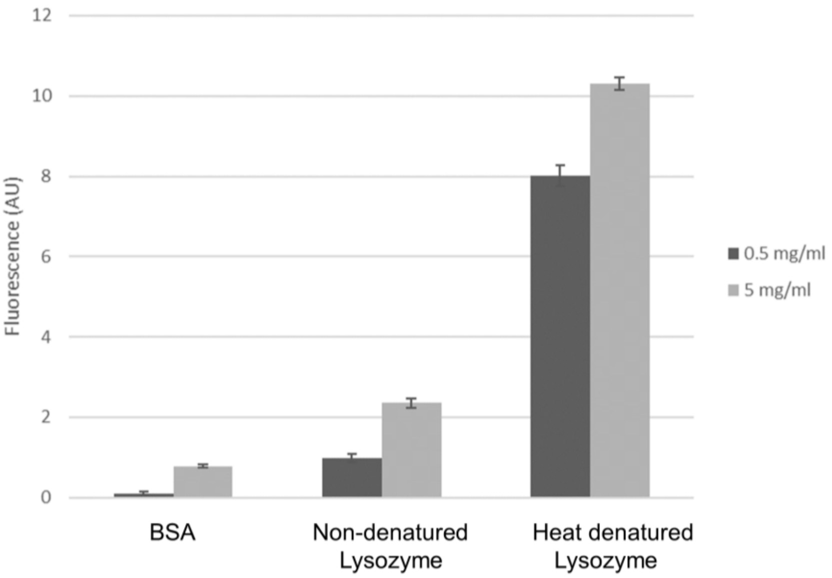

Figure S3: ThT assay controls: BSA and non-denatured lysozyme (negative control) and heat-denatured lysozyme were assayed at 5 mg/ml and 0.5 mg/ml. Results are presented in arbitrary units (AU) of fluorescence, as mean ± standard deviation; n = 3.
